# Oil Palm Phenolics Inhibit the* In Vitro* Aggregation of *β*-Amyloid Peptide into Oligomeric Complexes

**DOI:** 10.1155/2018/7608038

**Published:** 2018-01-31

**Authors:** Robert P. Weinberg, Vera V. Koledova, Hyeari Shin, Jennifer H. Park, Yew Ai Tan, Anthony J. Sinskey, Ravigadevi Sambanthamurthi, ChoKyun Rha

**Affiliations:** ^1^Biomaterials Science and Engineering Laboratory, Massachusetts Institute of Technology, Cambridge, MA 02139, USA; ^2^Malaysian Palm Oil Board, No. 6, Persiaran Institusi, Bandar Baru Bangi, 43000 Kajang, Selangor, Malaysia; ^3^Department of Biology, Massachusetts Institute of Technology, Cambridge, MA 02139, USA

## Abstract

Alzheimer's disease is a severe neurodegenerative disease characterized by the aggregation of amyloid-*β* peptide (A*β*) into toxic oligomers which activate microglia and astrocytes causing acute neuroinflammation. Multiple studies show that the soluble oligomers of A*β*42 are neurotoxic and proinflammatory, whereas the monomers and insoluble fibrils are relatively nontoxic. We show that A*β*42 aggregation is inhibited* in vitro* by oil palm phenolics (OPP), an aqueous extract from the oil palm tree* (Elaeis guineensis)*. The data shows that OPP inhibits stacking of *β*-pleated sheets, which is essential for oligomerization. We demonstrate the inhibition of A*β*42 aggregation by (1) mass spectrometry; (2) Congo Red dye binding; (3) 2D-IR spectroscopy; (4) dynamic light scattering; (5) transmission electron microscopy; and (6) transgenic yeast rescue assay. In the yeast rescue assay, OPP significantly reduces the cytotoxicity of aggregating neuropeptides in yeast genetically engineered to overexpress these peptides. The data shows that OPP inhibits (1) the aggregation of A*β* into oligomers; (2) stacking of *β*-pleated sheets; and (3) fibrillar growth and coalescence. These inhibitory effects prevent the formation of neurotoxic oligomers and hold potential as a means to reduce neuroinflammation and neuronal death and thereby may play some role in the prevention or treatment of Alzheimer's disease.

## 1. Introduction

Alzheimer's disease (AD) is an aging-associated, progressive, debilitating neurodegenerative disorder of the brain whose pathogenesis is associated with the aggregation of the amyloid-*β* peptide (A*β*42/A*β*40) [1–5]. The A*β*42,/A*β*40 aggregates form soluble oligomers and insoluble fibrillar deposits extraneuronally resulting in the activation of multiple pathophysiological processes with substantial loss of synapses and neurons [6–8]. Current evidence strongly supports the role of misfolded proteins forming amyloid aggregates playing a role in the pathogenesis of several diseases including Alzheimer's, Parkinson's, and Huntington's diseases as well as type II diabetes mellitus [9–12].

The amyloid-*β* aggregates into soluble neurotoxic oligomers [13–16] in a well-characterized fibril oligomerization process eventually forming stacked beta-sheet-rich fibrils [17–22]. The amyloid-*β* fibrils have a common parallel, in-register stacking organization based on the beta-pleated sheet stacking of the amyloid-*β* peptide [16, 23–26]. Whereas the monomers are relatively nontoxic, the neurotoxic oligomers may induce pathogenic pores in neurons [27, 28], create abnormal calcium ion fluxes [29] altering intracellular signaling pathways including MAPK, along with increased oxidative stress [29–32], and also activate microglia and astrocytes to an acute inflammatory state releasing such cytokines as TNF-*α*, IL-1b, and IFN-*γ* [33, 34].

The A*β* peptide-induced neuroinflammatory state results in synaptic loss and neuronal death in the brain regions essential for cognitive function, including the hippocampus, entorhinal cortex, and cerebral cortex [35]. This pathology results in the loss of memories, personalities, and cognitive function resulting in dementia.

A*β* is a 4 kDa peptide with several isoforms, the most common being a 40- and 42-amino acid peptide (A*β*40/A*β*42) [35]. The A*β* is a 39–44 amino acid peptide [17, 36–40] which is endoproteolytically cleaved from a cell surface protein, the amyloid precursor protein (APP), by *α*-, *β*-, and *γ*-secretases [41]. At a critical molar concentration, A*β* undergoes an extensively studied 3-phase accretion into fibrils involving a nucleation-elongation process: lag phase, exponential growth, and plateau phase [5, 42–54]. The A*β* fibrils have a parallel in-register structure [26]. The A*β* fibrils and the oligomerization process [55–58] have been well characterized by multiple physicochemical measurements including transmission electron microscopy (TEM) [59], X-ray diffraction [60, 61], mass spectrometry (MS) [62–64], 2D infrared spectroscopy (2D-IR), circular dichroism (CD), Congo Red dye binding (CR), Thioflavin-T fluorescence (TTf) [65, 66], SDS-gel electrophoresis (SDS-PAGE) [67], atomic force microscopy (AFM) [68, 69], gel filtration [70, 71], fluorescence resonance energy transfer (FRET) [72, 73], dynamic light scattering (DLS) [74], Fourier-transform infrared spectroscopy (FTIR), and nuclear magnetic resonance imaging (NMR) [75]. The soluble spherical oligomer aggregates measure approximately 2–20 nm [68, 69, 76, 77] by hydrodynamic radius (DLS) in aqueous solution.

Initially the amyloid-*β* peptide forms dimers in aqueous solution following a lag phase involving a nucleation event [70, 71]. The gradual accumulation of dimers leads to the formation of higher order aggregates [78]. The molecular weights of these higher order aggregates range between 10^5^ and 10^6^ Daltons (Da) [71, 79] comprising spherical particles with an average diameter of 3 nm [59, 76, 77] with an average of 24 A*β* monomers per particle. The aggregation into higher order oligomers requires a minimum critical concentration of 25 *μ*M A*β*. Following further incubation, the spherical particles coalesce to form curvilinear fibrils which have a characteristic bearded appearance, called protofibrils [59, 68, 69, 78]. In a continuing process of coalescence, the growing mature A*β* fibrils consume the spherical aggregates and protofibrils which disappear from solution [80].

The neurotoxic oligomers are A*β* aggregates which do not precipitate at 100,000 ×g centrifugation [81–83]. These soluble A*β* oligomers correlate better with AD symptomatology and dementia than the fibrillar A*β* [84, 85]. There are cognitively normal persons with higher numbers of fibrillar A*β* plaques. There is a poor correlation between the presence of fibrillar amyloid deposits and dementia symptomatology [35]. The modified “amyloid hypothesis” of AD states that the soluble A*β* oligomers are neurotoxic, but the fibrillar A*β* deposits are not toxic [86–89]. Multiple studies of A*β* oligomers show that they are toxic for neurons* in vitro* [86, 90, 91].

## 2. Materials and Methods

### 2.1. Preparation of Oil Palm Phenolics

The preparation of oil palm phenolics (OPP) has been previously described [92, 93]. OPP is an aqueous solution extracted from the fruit of the palm oil tree* (Elaeis guineensis)*. Following harvesting, the oil palm fruit is mechanically crushed and squeezed under high pressure steam. The biphasic filtrate is then decanted to separate the top oil layer from the bottom aqueous layer. This aqueous layer is now termed “oil palm phenolics” and contains such phytochemicals as polyphenols, flavonoids, phenolic acids, shikimate, oligosaccharides, and metal ions.

### 2.2. A*β*42 Amyloid Peptide

The A*β*42 was purchased as a lyophilized powder comprising 0.25 mg of purified A*β*42 from Millipore (Waltham, Massachusetts, USA). Then the 250 mcg A*β*42 was initially dissolved in 50 microliters of DMSO (dimethyl sulfoxide). This 50-microliter solution was then mixed with 950 microliters of phosphate-buffered saline (PBS, 10 mM, pH 7.4). This solution was aliquoted and frozen at −20°C. Assays were carried out at a final concentration of 50 *μ*M A*β*42 in PBS (pH 7.4). A*β*42 aggregation was effected by incubating the solution at 30°C., without stirring, for the indicated time periods.

### 2.3. Congo Red Dye Binding Assay

The Congo Red dye working solution was prepared as a 20 *μ*M Congo Red (Sigma Aldrich) in phosphate-buffered saline (PBS, pH 7.4). The Congo Red dye binding assays were performed with a TECAN Spectrafluor Plus instrument using 96-well plastic microtiter plates (BD Falcon). The stock 50 *μ*M A*β*42 solution was diluted with PBS to a final concentration of 10 *μ*M A*β*42. Then 50 *μ*L of this 10 *μ*M A*β*42 solution was added to each of the wells of the 96-well microtiter plate. Following this, 50 *μ*L of OPP, at the appropriate concentrations, was added to each well. Finally 6 *μ*L of the 20 *μ*M Congo Red dye solution was added to each well. The TECAN Spectrafluor was set to take six repeat optical absorption readings at each time interval of 15 minutes, with absorption readings taken at the wavelengths of 492 nm and 540 nm. The optical absorption readings at these two wavelength allowed calculation of the A*β*42 aggregates because of the known spectral shift which occurs when Congo Red is bound to aggregated A*β*42 (see (1) below). The TECAN Spectrafluor was set for incubation of plates at 30°C. with time intervals set at optical absorption measurements taken every 15 minutes.

Aggregated A*β*42 may be calculated by the following equation:

(1) $$\begin{array}{c} C_{b}=\frac{A_{541}}{47\text{,}800}-\frac{A_{403}}{38\text{,}100}, \end{array}$$

where *C*_*b*_ represents the amount of Congo Red dye bound to aggregated peptide, *A*_541_ is the optical absorption measured at the wavelength of 541 nm, *A*_403_ is the optical absorption measured at the wavelength of 403 nm, 478000 is the extinction coefficient at 541 nm, and 38100 is the extinction coefficient at 403 nm.

### 2.4. Thioflavin-T Fluorescence Assay

Although much work has been done with Thioflavin-T to study the kinetics of aggregation and fibrillization, we are limited in the use of this dye because of the very strong fluorescent signal emitted by the oil palm phenolics at the wavelength of 535 nm, following excitation at a wavelength of 450 nm. The OPP fluorescent signal is 4-5 times greater than that of Thioflavin-T bound to aggregates. Therefore, the OPP signal completely masks any detectable signal from Thioflavin-T.

### 2.5. Mass Spectrometry by MALDI-TOF

Ab42 MALDI-TOF mass spectrometric analyses were performed using a Microflex mass spectrometer (Bruker Daltonics, Billerica, MA, USA) equipped with a pulsed nitrogen laser operating at 337 nm. Small oligomer positive ion spectra were acquired in linear mode over an *m*/*z* range from 2000 to 50,000 using a 20-kV accelerating voltage and a 150-ns delay extraction time. The spectrum for each spot was obtained by averaging the results of 200 laser shots. The analysis was performed by spotting on the target plate 1.0 ul of the sample mixed with an equal volume of the matrix solution, 10 mg/ml sinapinic acid (Sigma Aldrich), in CH3CN/H2O (50 : 50, v/v) containing 0.1% (v/v) trifluoroacetic acid (Sigma Aldrich). The sample was prepared in the following way: 10 microliters was C4 Zip-Tipped, eluted in 1 microliter of 70% acetonitrile, mixed with 1 microliter of matrix, spotted, and allowed to air dry.

### 2.6. Transmission Electron Microscopy (TEM)

Copper grids with Formvar carbon coating (400 meshes, Ted Pella) were glow discharged for 20 seconds and 5 *μ*l of amyloid sample was placed on the grids for 5 minutes. The excess sample on the grids was blotted off using filter paper. The grids were then floated onto a drop of filtered 1.5% uranyl acetate (Sigma Aldrich) for 45 seconds. Then the sample grids are placed under a JEOL 1200 SX Transmission Electron Microscope (TEM) and digital photomicrographs were taken using the AMT 16000 camera system.

### 2.7. 2D FTIR Assay

Similar to NMR spectroscopy, two-dimensional infrared spectroscopy (2D-IR) provides important information on the secondary structure of polypeptides including the quantification of alpha-helices and beta-pleated sheets at high sensitivity as developed in the MIT laboratory of Professor Andrei Tokmakoff [94–98]. This secondary structural information on the polypeptide is obtained by spreading the observed spectral information onto two frequency axes and correlating the observed frequency of initial vibrational excitation (*ω*_1_) with the final observed detection frequency (*ω*_3_).

Tokmakoff has demonstrated that the observed frequencies of the diagonal peaks on such a plot correspond to the vibrational transitions within the sample, and cross-peaks can only be observed when two vibrational modes are coupled (i.e., if the modes reside within the same structure or if there is energy transfer between two vibrations). Thus, in a 2DIR spectrum, each positive diagonal peak is accompanied by a negative appearing below the diagonal; these negative peaks are from vibrational transitions involving two-quantum states and contain information related to the anharmonicities of the individual modes. Correlation 2DIR spectra were acquired using a Fourier-transform 2D-IR spectrometer described in detail elsewhere.

Amyloid-beta samples were prepared in D2O at a concentration of 10 mg/ml and buffered to a final pH of 7.4 in a 10 mM deuterated phosphate and held between CaF2 windows in a 50 um path-length cell. Spectra were collected in the perpendicular (ZZYY) polarization geometry to enhance the intensity of the cross-peak.

### 2.8. Dynamic Light Scattering (DLS)

In order to determine the size of the A*β*42 peptide aggregates in the presence and absence of OPP, dynamic light scattering (DLS) measurements were performed. Dynamic light scattering (DLS) was performed with a DynaPro Plate Reader (Wyatt Technology, Santa Barbara, California) using Wyatt optically transparent 96-well microtiter plates. The sensitivity of the DynaPro Plate Reader is able to measure protein aggregates with a hydrodynamic radius between 0.5 and 1000 nm and weight-average molar masses between 1 and 1000 kDa. Incubation temperature of the plates was maintained at 30°C. during aggregation studies.

The measured hydrodynamic radius (Rh) was the average of 50 measurements. The mean Rh and polydispersity (Pd) were estimated, on the basis of an autocorrelation analysis of scattered light intensity based on the translational diffusion coefficient, from the Stokes-Einstein equation:

(2) $$\begin{array}{c} R_{h}=\frac{kT}{6\pi \eta D}, \end{array}$$

where *R*_*h*_ is the hydrodynamic radius (nm), *k* is Boltzmann's constant, *T* is the absolute temperature (K), *η* is the viscosity of water, and *D* is the translational diffusion coefficient [99]. The stock 50 *μ*M A*β*42 solution was diluted with PBS to a final concentration of 10 *μ*M A*β*42. Then 50 *μ*L of this 10 *μ*M A*β*42 solution was added to each of the wells of the 96-well microtiter plate. Following this, 50 *μ*L of the diluted OPP was added to each well, at concentrations between 0 and 8 mcg/ml (0, 2, 4, 6, and 8).

### 2.9. Transgenic Yeast Rescue Assay

Yeast cells were genetically engineered using transduced nucleic acid sequences to overexpress neuropeptides in the Lindquist lab at MIT as previously described [100–103]. Yeast cells were grown in rich media (YPD) or in synthetic media lacking uracil and containing 2% glucose (SD/-Ura), raffinose (SRaf/-Ura), or galactose (SGal/-Ura). Gateway entry clones containing the full-length neuropeptides (*β*-amyloid, *α*-synuclein, Huntingtin, and TDP-43) in the vector pDONR221 were obtained from Invitrogen. A Gateway LR reaction was used to shuttle each neuropeptide into Gateway-compatible yeast expression vectors (pAG vectors, http://www.addgene.org/kits/lindquist-yeast-gateway/). To generate C-terminally GFP-tagged neuropeptide constructs, a two-step PCR protocol was used to amplify the neuropeptide sequence without a stop codon and incorporate the Gateway attB1 and attB2 sites along with a Kozak consensus sequence. Resulting PCR products were shuttled into pDONR221 using a Gateway BR reaction. The entry clones (*β*-amyloid_nostop_, *α*-synuclein_nostop_, Huntingtin_nostop_, and TDP-43_nostop_) were then used in LR reactions with pAG426Gal-ccdB-GFP to generate the 2 *μ*m neuropeptide-GFP fusion constructs. To generate the integrating neuropeptide-GFP construct, the neuropeptide_nostop_ entry clone was used in an LR reaction with pAG306Gal-ccdB-GFP. Two-micron plasmid constructs (e.g., pAG426Gal-TDP-43-GFP) were transformed into BY4741* (MATa his3 leu2 met15 ura3)*. The neuropeptide-GFP integrating strain was generated by linearizing pAG306Gal-neuropeptide-GFP by Bsm1 restriction digest, followed by transformation in the w303 strain* (MATa can1-100, his3-11,15, leu2-3,112, trp1-1, ura3-1, and ade2-1)*. The* hsp104Δ* and* rnq1Δ* strains are deletion mutants (gene disrupted by KanMX4) obtained from the haploid deletion collection (Invitrogen).

Yeast procedures were performed according to standard protocols. The PEG/lithium acetate method was used to transform yeast with plasmid DNA. For spotting assays, yeast cells were grown overnight at 30°C. in liquid media containing raffinose (SRaf/-Ura) until they reached log or mid-log phase. Cultures were then normalized for OD600, serially diluted, and spotted onto synthetic solid media containing glucose or galactose lacking uracil and were grown at 30°C. for 2-3 days.

As the yeast grow, synthesis of increasing levels of the neuropeptide resulted in the formation of cytotoxic aggregates which severely limited yeast growth eventually killing the yeast, unless a rescue drug was administered which would prevent the aggregated neuropeptide cytotoxicity. The transgenic yeast growth data is expressed as percentage growth relative to the control strain, which accounts for some values being greater than 100%.

### 2.10. Statistical Analysis of Data

All data were presented as mean ± standard deviation from 3 independent measurements. The statistical analysis was made by performing one-way ANOVA for the 3 independent determinations. Significance of results was determined as *p* ≤ 0.01 [unless otherwise stated].

## 3. Results

### 3.1. Congo Red Dye Binding

**Figure 1:**
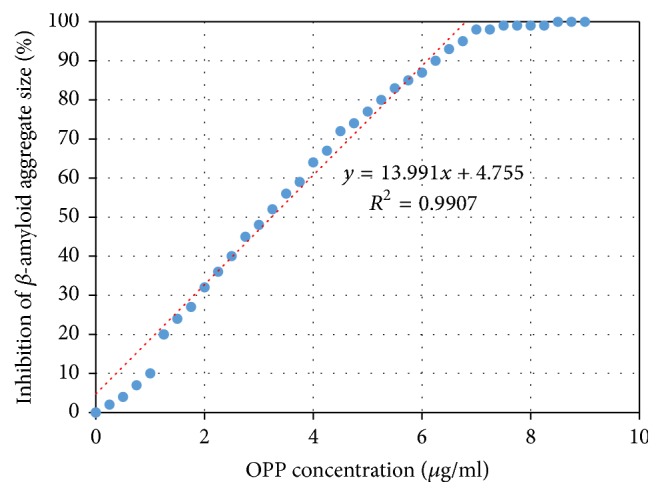
Spectral compensated curve for Congo Red dye binding. Dose-dependent inhibition of A*β*42 aggregation by OPP. Equation (1) was used to quantify the aggregates from the spectral shift between wavelengths 403 nm and 541 nm. IC50 = 3.24 *μ*g/ml.

The Congo Red binding assay is one of the earliest methods developed to measure the aggregation of A*β*42. The Congo Red assay is based upon a spectral shift which occurs in the absorption of Congo Red at 2 different reference wavelengths when the Congo Red is bound to beta amyloid peptide monomers versus aggregates. The aggregation data in Figure 1 is normalized with the maximum inhibition of oligomerization set equal to 100% inhibition.

As seen in Figure 1, increasing concentrations of OPP result in an increased inhibition of A*β*42 aggregation. We see that the concentration-inhibition relationship is somewhat linear between OPP concentrations of 1.0 and 7.0 *μ*g/ml. Also it appears that maximum aggregation inhibition occurs at an OPP concentration of 7.0 *μ*g/ml.

Based on the above OPP inhibition of A*β*42 aggregation, the IC50 (50% inhibitory concentration) is 3.24 *μ*g/ml OPP.

### 3.2. Dynamic Light Scattering

**Figure 2:**
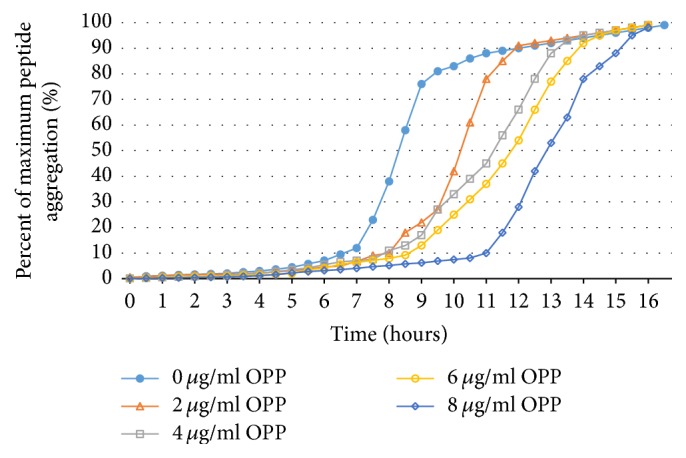
DLS kinetics of A*β* aggregation inhibition by OPP. This figure reveals both the dose-dependent and time-dependent effects of inhibitory effects of OPP on A*β*42 aggregation. Doses vary between 0 and 8 *μ*g/ml OPP with kinetics studied over a 16-hour period of time.

In Figure 2, we see that increasing the concentration of OPP results in prolonging the lag phase of aggregation. Generally the exponential phase of aggregate growth has a similar sigmoidal curve at the concentrations tested. The inhibition of A*β*42 aggregation by OPP reveals both a dose-dependent and a time-dependent component.

### 3.3. Mass Spectrometry

**Figure 3:**
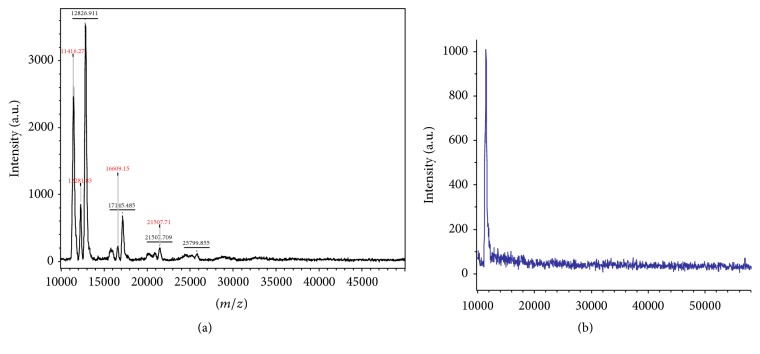
Mass spectrometric display of A*β*42 aggregate size. (a) A*β*42 in PBS (negative control); (b) A*β*42 with 10 *μ*g/ml OPP. (a) shows the presence of monomers, dimers, trimers, tetramers, and pentames. (b) Shows the presence of only monomers.

In Figure 3(a), in the absence of OPP, we see A*β*42 aggregate peaks representing dimers, trimers, tetramers, hexamers, and septamers. In Figure 3(b), in the presence of OPP, only the monomeric A*β* is seen at MW of 4,500 Da. The mass spectroscopy data clearly shows that the presence of OPP will inhibit the aggregation and polymerization of beta amyloid peptide. At a concentration of 10 *μ*g/ml OPP, only the monomeric A*β*42 is present in solution.

### 3.4. Tabulation of Mass Spectrometric Data Pooled from 5 MALDI-TOF Runs

**Figure 4:**
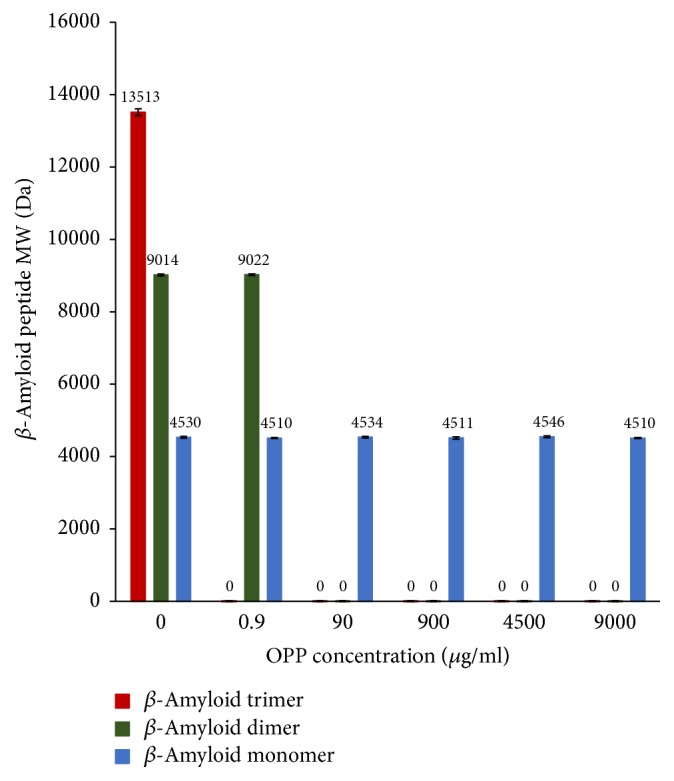
MALDI-TOF mass spectrometric data on A*β*42 aggregation pooled from 5 runs. In the absence of OPP (0 *μ*g/ml), A*β*42 dimers and trimers form. At an OPP concentration of 0.9 *μ*g/ml, only dimers will form. At 90 *μ*g/ml and higher, only monomers exist and no dimers nor trimers are formed.

In Figure 4, we see the pooled data for 5 mass spectrometric runs. In the absence of OPP (0 mcg/ml), we see that there are 3 species of the peptide found: the monomer, the dimer, and the trimer. At a concentration of 0.9 *μ*g/ml OPP, we see that only 2 species are found: the monomer and the dimer. Then from a concentration of 90 *μ*g/ml up to 9 mg/ml, we see that only the monomeric form of the beta amyloid peptide is found.

### 3.5. 2D-IR Determinations of Secondary Polypeptide Structure

**Figure 5:**
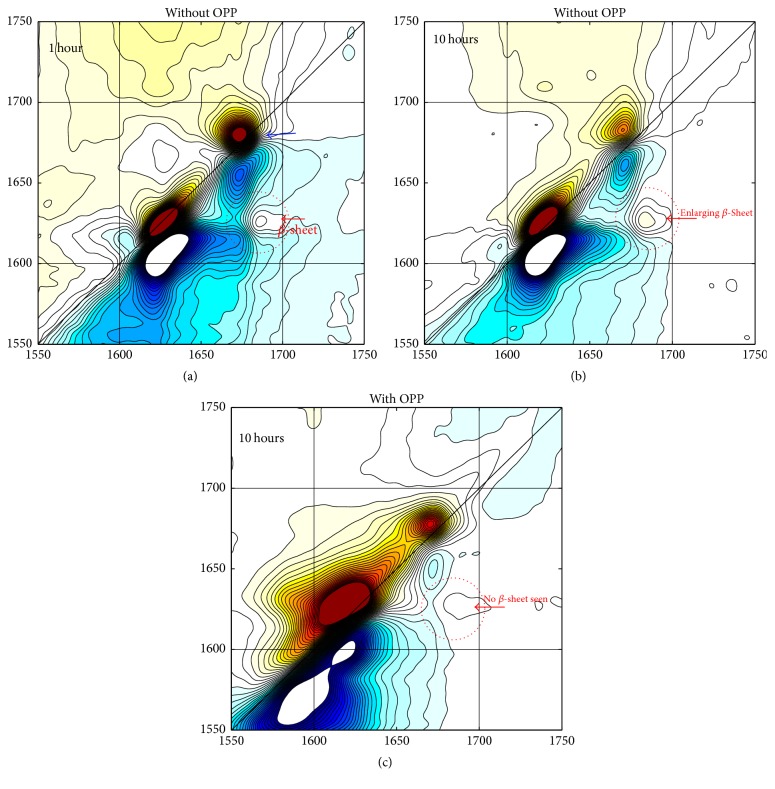
Amide-I two-dimensional correlation spectra of the A*β*42 samples incubated at 37°C. without OPP at 1-hour (a) and at 10-hour (b) incubation. A*β*42 sample incubated at 37°C. with 10 *μ*g/ml OPP at 10 hours (c). The diagonal peak at 1672 cm^−1^, indicated by a blue arrow, is due to tetrafluoroacetic acid (TFA) present in the sample. The cross-peak near [*ω*1, *ω*3] = [1620,1680] cm^−1^.

Figure 5 represents the amide-I two-dimensional IR correlation spectra of the A*β*42 showing the frequency plots in the amide-I region, where the beta-sheets are characterized by the presence of two peaks centered near 1620 and 1680 cm^−1^, whose individual amide oscillators vibrate in-phase perpendicular or parallel to the *β*-strands, respectively. The splitting between these modes at this frequency is related to the size of the folded *β*-sheet. In a primarily beta-sheet protein, the corresponding cross-peaks give a characteristic Z-shape to the spectrum. Here the focus is primarily on the cross-peak centered at [*ω*1, *ω*3] = [1620,1680] cm^−1^ whose amplitude is indicative of the total amount of beta-sheet present in the sample.

Three 2D-IR spectra are shown in Figure 5; these correspond to the amyloid-beta sample incubated for a period of 1 hour (a) and 10 hours (b) at 37°C in the absence of OPP, and at 10 hours in the presence of 10 *μ*g/ml OPP (c).

The spectra in (a) and (b) show that the cross-peak at [*ω*1, *ω*3] = [1620,1680] cm^−1^ (indicated by a red arrow) increases in amplitude over this period. This is indicative of an overall increase in beta-sheet content of the sample; we ascribe this increase in amplitude to growth of the beta-fibrils in the sample. Longer incubation times do not affect amplitude of the cross-peak (data not shown).

The sample incubated with 10 *μ*g/ml OPP (c) shows a very small cross-peak even after incubation for 10 hours. Furthermore, the diagonal peaks associated with beta-sheet become significantly broader and there is an increase in signal near the 1650 cm^−1^ region, a part of the spectrum associated with helical and random-coil conformations, suggesting that OPP induces disorder within the secondary structure of the amyloids and therefore prevents the formation of beta-fibrils.

### 3.6. A*β*42 Electron Micrographs from Transmission Electron Microscopy (TEM)

In the transgenic yeast assay, the presence of 10 *μ*g/ml OPP rescues the growth of the *β*-amyloid-producing yeast from 20% (without OPP) to 40% (with OPP). For the Huntingtin-producing yeast, 10 *μ*g/ml OPP rescues the yeast growth from 25% (without OPP) to 60% (with OPP). For the TDP-43-producing yeast, 10 *μ*g/ml OPP rescues the yeast growth from 25% (without OPP) to 190% (with OPP). We see that there is no significant change of growth for the *α*-synuclein-producing yeast with or without OPP. These relative growth percentages compare the transgenic yeast growth to growth of control strains (Figure 7).

## 4. Discussion

Since the discovery that the beta amyloid peptide (A*β*) is the primary constituent of the fibrils found in the extraneuronal senile neuritic plaques in the brains of Alzheimer's patients, this peptide has played a central role in Alzheimer's research. It is now believed that the soluble oligomers are neurotoxic and the mature fibrils of A*β* peptide are not neurotoxic per se. Some speculate that the mature amyloid fibrils may serve as a reservoir of soluble oligomers of A*β*. There is experimental evidence that larger aggregates may reversibly release smaller aggregates following oligomerization. The soluble A*β* oligomers are defined as what remains in aqueous solution following the high-speed centrifugation of brain extracts. The 2 most common human isoforms of A*β* are A*β*40 [40 amino acids in length] and A*β*42 [42 amino acids in length]. A*β*42 peptide is known to be more fibrillogenic than A*β*40 so we have carried out these aggregation studies with A*β*42.

The data presented herein show that OPP significantly inhibits the oligomerization of A*β*42, in part by preventing beta-sheet folding of the peptide. Furthermore, OPP also reduces the cytotoxicity of aggregated neuropeptides of A*β*42, Huntingtin, and TDP-43 peptides in transgenic yeast.

The Congo Red data shows a linear relationship between the degree of aggregation inhibition and the concentration of OPP over the range of 1.0–7.0 *μ*g/ml OPP. There is 50% inhibition of A*β* aggregation at an OPP concentration of 3.24 *μ*g/ml OPP (IC50) (Figure 1). Dynamic light scattering shows successive prolongation of the lag phase at higher concentrations of OPP (Figure 2). The phase of exponential fibrillar growth shows a lower slope at higher concentrations of OPP. Mass spectrometry shows distinct peaks for the monomer, dimer, trimer, tetramer, pentamer, hexamer, and septamer in A*β*42 solution without OPP (Figure 3(a)). Mass spectrometry shows a single low-molecular weight peak in the A*β*42 solution with OPP (Figure 3(b)). The data for multiple mass spectrometry runs are pooled in Figure 4. The A*β*42 trimers only form reproducibly in the absence of OPP. A*β*42 dimers form at a concentration of 0.9 mcg/ml OPP and in the absence of OPP. Only A*β*42 monomers are consistently seen at OPP concentrations of 90, 900, 4,500, and 9,000 mcg/ml.

**Figure 6:**
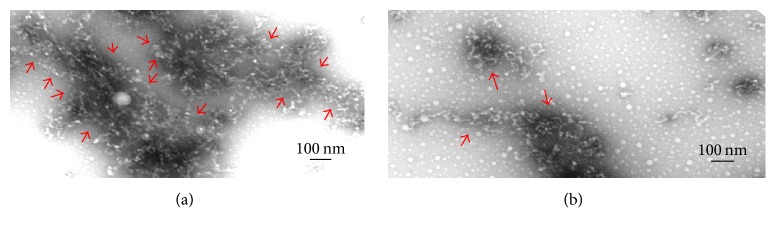
TEM of *β*-amyloid. (a) *β*-Amyloid (PBS); (b) *β*-amyloid (10 *μ*g/ml OPP). (a) shows the large A*β*42 aggregates and meshes (size greater than 10 nm) which form in the absence of OPP. (b) shows the smaller aggregates which are more sparse which form in the presence of 10 *μ*g/ml OPP.

**Figure 7:**
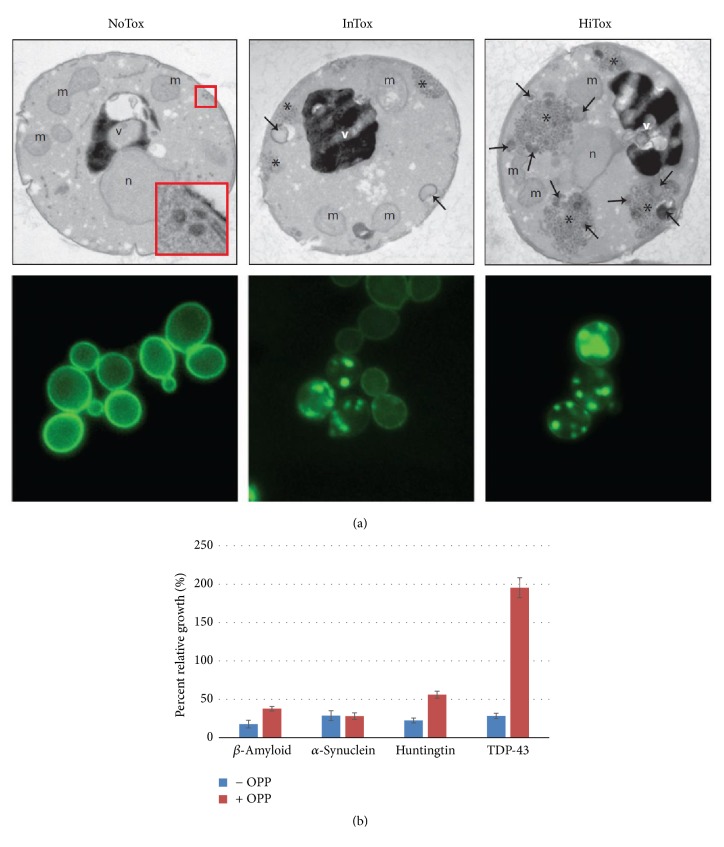
(a) Transgenic yeast which overexpresses the TDP-43 peptide. NoTox = low expression, TDP-43; IntTox = intermediate expression, TDP-43 aggregation seen as clumps of GFP-TDP43; HiTox = high expression, TDP-43 in larger clumps and increased death of yeast. Arrows demarcate the inclusion granules of TDP-43 (Susan Lindquist, MIT). (b) OPP significantly reduces the cytotoxicity of the aggregated neuropeptides in the transgenic yeast rescue assay. Bar heights represent percent growth relative to control strain. OPP doubles the growth for *β*-amyloid-producing yeast from 20% (without OPP) to 40% (with OPP); more than doubles the growth for Huntingtin-producing yeast from 25% (without OPP) to 60% (with OPP). OPP increases the growth of TDP-43-producing yeast more than 6-fold from 25% (without OPP) to 190% (with OPP). There is no significant change in the growth of the *α*-synuclein-producing yeast with or without OPP. OPP dose was 10 *μ*g/ml for the 4 peptide-producing transgenic yeasts.

2D-IR spectroscopy shows a developing beta-sheet signal as early as 1 hour with significant increase in size of that signal by 10 hours in the absence of OPP (Figure 5). At an OPP concentration of 10 mcg/ml, no beta-sheet signal is seen after 10 hours of incubation. Following 20 hours of incubation at 30°C. in the presence of 10 *μ*g/ml OPP, few small aggregates of A*β*42 can be visualized using transmission electron microscopy (TEM) (Figure 6(b)). However, multiple large aggregates of A*β*42 can be visualized by TEM when the A*β*42 was incubated in the presence of 15 *μ*g/ml OPP (Figure 6(a)).

In the transgenic yeast assay, the presence of 10 *μ*g/ml OPP rescues the growth of the *β*-amyloid-producing yeast from 20% (without OPP) to 40% (with OPP). For the Huntingtin-producing yeast, 10 *μ*g/ml OPP rescues the yeast growth from 25% (without OPP) to 60% (with OPP). For the TDP-43-producing yeast, 10 *μ*g/ml OPP rescues the yeast growth from 25% (without OPP) to 190% (with OPP). We see that there is no significant change of growth for the *α*-synuclein-producing yeast with or without OPP. These relative growth percentages compare the transgenic yeast growth to growth of control strains.

A*β*_40_ and A*β*_42_ are the most common human isoforms of the peptide; A*β*_40_ is the more common isoform, but A*β*_42_ is more fibrillogenic, aggregates more quickly, and appears to be the more toxic isoform, especially in the dimer state. A*β* has little toxicity while in the monomeric state. Based upon neurotoxicity data* in vitro*, animal models, and clinical observations in patients with Alzheimer's disease, one therapeutic goal consists of inhibiting the formation of soluble A*β* dimers, thus nullifying the neurotoxic effect of these dimers and should be effective in preventing and/or treating Alzheimer's disease. Statistical studies on patients with AD have shown that the cortical levels of soluble A*β* correlate well with both the extent of synaptic loss and the severity of the clinical symptoms [81, 104, 105]. Multiple studies have shown that the soluble assemblies of oligomeric A*β* are the neurotoxic species which cause the cognitive losses of Alzheimer's disease [16, 23–26]. These oligomeric A*β* (oA*β*) have also been called A*β*-derived diffusible ligands (ADDLs). Recent research has shown that the oA*β*/ADDLs play a key role in cognitive decline [27, 28].

The “amyloid hypothesis” that small soluble oligomers of A*β* underlie the key phenotypic characteristics of Alzheimer's disease is supported by experimental data showing that these small soluble oligomers may (1) cause synaptic loss and decreased dendritic spine density; (2) cause hyperphosphorylation of tau proteins with resulting intraneuronal neurofibrillary tangles and collapse of the neuritic cytoskeleton; and (3) cause memory impairment and cognitive losses in the absence of amyloid plaques.

The experimental evidence presented in this paper shows that oil palm phenolics, derived from the palm oil plant* (Elaeis guineensis)*, have inhibitory effects on the process of aggregation and polymerization of beta amyloid peptide* in vitro*. This inhibition may cause the beta amyloid peptide to remain in the soluble monomeric state and facilitate the clearance of the peptide from the brain via the normal physiologic mechanisms. Several pharmaceutical companies are currently developing drugs which inhibit the aggregation and oligomerization of A*β*42. Preventing the formation of neurotoxic oligomers may represent a potential preventive or therapeutic strategy in the treatment of Alzheimer's disease.

Thus, the inhibitory effects of OPP on A*β*42 aggregation may lead to the development of a potential drug for the prevention and/or treatment of Alzheimer's disease. Our next step consists in identifying, isolating, and purifying that component(s) of the oil palm phenolics which has these inhibitory and antifibrillogenic effects on beta amyloid peptide.

## 5. Conclusions

This is a study of the aggregation of *β*-amyloid peptide* in vitro* and the inhibitory effects of OPP on this aggregation. The study led to the following experimental results:

1. Oil palm phenolics inhibit *β*-amyloid monomers from aggregating into dimers, trimers, or larger aggregates as shown by the observed reduction in aggregate size as measured by Congo Red binding, mass spectrometry, dynamic light scattering, 2D-IR spectroscopy, and transmission electron microscopy.
2. Oil palm phenolics prolong the lag phase in the nucleation-elongation process of A*β*42 oligomerization as observed by dynamic light scattering with a shift of the aggregation curves to the right.
3. Oil palm phenolics inhibit the folding of the beta-pleated sheet as observed in the 2D infrared spectroscopy.
4. Fifty percent (50%) of the maximum aggregate size is observed at an OPP concentration of 3.24 *μ*g/ml (IC50) by Congo Red dye binding.
5. At an OPP concentration greater than or equal to 90 *μ*g/ml, only monomers of A*β*42 are observed by mass spectrometry. No dimers, trimers, or higher oligomers are observed at this concentration or higher.
6. The cytotoxicity of aggregated neuropeptides is greatly reduced by OPP at a concentration of 10 *μ*g/ml in transgenic yeast which overexpresses these peptides.

The current scientific literature shows that the soluble A*β*42 oligomers are neurotoxic and cause extensive pathologic changes in neurons, decrease dendritic spine density, and cause depression of long-term potentiation in neurons and enhancement of long-term depression. Therefore, the properties of the oil palm phenolics to inhibit the formation of oligomers may hold promise for the medical treatment and/or prevention of Alzheimer's disease.
